# Approaches for the Use of AI in Workplace Health Promotion and Prevention: Systematic Scoping Review

**DOI:** 10.2196/53506

**Published:** 2024-08-20

**Authors:** Martin Lange, Alexandra Löwe, Ina Kayser, Andrea Schaller

**Affiliations:** 1 Department of Fitness & Health IST University of Applied Sciences Duesseldorf Germany; 2 Department of Communication & Business IST University of Applied Sciences Duesseldorf Germany; 3 Institute of Sport Science, Department of Human Sciences University of the Bundeswehr Munich Munich Germany

**Keywords:** artificial intelligence, AI, machine learning, deep learning, workplace health promotion, prevention, workplace health promotion and prevention, technology, technologies, well-being, behavioral health, workplace-related, public health, biomedicine, PRISMA-ScR, Preferred Reporting Items for Systematic Reviews and Meta-Analyses Extension for Scoping Reviews, WHPP, risk, AI-algorithm, control group, accuracy, health-related, prototype, systematic review, scoping review, reviews, mobile phone

## Abstract

**Background:**

Artificial intelligence (AI) is an umbrella term for various algorithms and rapidly emerging technologies with huge potential for workplace health promotion and prevention (WHPP). WHPP interventions aim to improve people’s health and well-being through behavioral and organizational measures or by minimizing the burden of workplace-related diseases and associated risk factors. While AI has been the focus of research in other health-related fields, such as public health or biomedicine, the transition of AI into WHPP research has yet to be systematically investigated.

**Objective:**

The systematic scoping review aims to comprehensively assess an overview of the current use of AI in WHPP. The results will be then used to point to future research directions. The following research questions were derived: (1) What are the study characteristics of studies on AI algorithms and technologies in the context of WHPP? (2) What specific WHPP fields (prevention, behavioral, and organizational approaches) were addressed by the AI algorithms and technologies? (3) What kind of interventions lead to which outcomes?

**Methods:**

A systematic scoping literature review (PRISMA-ScR [Preferred Reporting Items for Systematic Reviews and Meta-Analyses extension for Scoping Reviews]) was conducted in the 3 academic databases PubMed, Institute of Electrical and Electronics Engineers, and Association for Computing Machinery in July 2023, searching for papers published between January 2000 and December 2023. Studies needed to be (1) peer-reviewed, (2) written in English, and (3) focused on any AI-based algorithm or technology that (4) were conducted in the context of WHPP or (5) an associated field. Information on study design, AI algorithms and technologies, WHPP fields, and the patient or population, intervention, comparison, and outcomes framework were extracted blindly with Rayyan and summarized.

**Results:**

A total of 10 studies were included. Risk prevention and modeling were the most identified WHPP fields (n=6), followed by behavioral health promotion (n=4) and organizational health promotion (n=1). Further, 4 studies focused on mental health. Most AI algorithms were machine learning-based, and 3 studies used combined deep learning algorithms. AI algorithms and technologies were primarily implemented in smartphone apps (eg, in the form of a chatbot) or used the smartphone as a data source (eg, Global Positioning System). Behavioral approaches ranged from 8 to 12 weeks and were compared to control groups. Additionally, 3 studies evaluated the robustness and accuracy of an AI model or framework.

**Conclusions:**

Although AI has caught increasing attention in health-related research, the review reveals that AI in WHPP is marginally investigated. Our results indicate that AI is promising for individualization and risk prediction in WHPP, but current research does not cover the scope of WHPP. Beyond that, future research will profit from an extended range of research in all fields of WHPP, longitudinal data, and reporting guidelines.

**Trial Registration:**

OSF Registries osf.io/bfswp; https://osf.io/bfswp

## Introduction

### Artificial Intelligence as an Umbrella Concept

Artificial intelligence (AI) is a concept that dates back to the mid-1900s [1] and was first defined as “the science and engineering of making intelligent machines” [2]. Today, AI is described as a computer system’s capability to perform complex tasks that mimic human cognitive functions to perform tasks such as reasoning, decision-making, or problem-solving, autonomously and adaptively [3]. However, its capabilities and underlying functions have changed significantly over the decades [1,4]. More recently, AI has emerged as a transformative force across various industries. Its application has shown promise in health promotion and health care [5-7], opening new possibilities concerning patient care and enhanced medical practices.

There is growing consensus in the literature that adaptivity and autonomy are the key characteristics of AI applications and technologies [5]. AI is considered an umbrella concept of emerging technologies, enclosing fundamental distinct types such as machine learning (ML), deep learning (DL), or natural language processing (NLP) [4,8]. Technically, AI is an ML-based approach that simulates human minds’ cognitive and affective functions [3,8] and is designed to observe and react to a specific environment. In contrast to deterministic programming, such models feature many free parameters that can adapt autonomously to calibrate the model. For example, AI can be applied in repetitive tasks requiring human intelligence, such as scanning and interpreting magnetic resonance imaging, autonomous driving, or analyzing big data sets [9-11]. ML and DL algorithms and artificial neural networks enable a machine or system to learn from large data sets, make autonomous decisions, and improve their performance over time [4]. More narrowly, NLP allows machines to generate and understand text and spoken language in the same way humans do. It combines rule-based natural language modeling with ML and DL models to process human language in text or speech data, understand its meaning, including feelings, and even generate human language, as it is sometimes used in chatbots or language translation [12].

### AI in Health Care and Public Health

Implementing AI algorithms and technologies for health care institutions bears enormous potential, ranging from efficient health service management, predictive medicine, patient data, and diagnostics with real-time analyses to clinical decision-making. Most studies report a broader AI architecture with a combination of algorithms rooted in ML, DL, and NLP [4,11]. For example, 1 AI approach evaluated the support of clinical decision-making by analyzing continuous laboratory data, past clinical notes, and current information of physicians synthesizing significant associations [13]. AI implementation in the form of predictive modeling showed positive results by detecting irregular heartbeats through smartwatches [14], automatically identifying reports of infectious disease in the media [15], or ascertaining cardiovascular risk factors from retinal images [16]. Through systematic profiling of 4518 existing drugs against 578 cancer cell lines with an AI-based approach, a study revealed that nononcology drugs have an unexpectedly high rate of anticancer activity [17]. Another study developed and evaluated a Medical Instructed Real-Time Assistant that listens to the user’s chief complaint and predicts a specific disease [18]. Chatbots have been used to detect COVID-19 symptoms through detailed questioning [6] or to predict the risk of type II diabetes mellitus [19].

### Workplace Health Promotion and Prevention

As adults spend a significant amount of time working, it is widely accepted that work and work environments have a major impact on individuals’ health. Workplace health promotion and prevention (WHPP) are important fields that “[…] improve the health and well-being of people at work […]” [20] through a combination of behavioral and organizational measures. Workplace health promotion follows a competence-oriented, salutogenetic approach to promoting the resources of an individual [20]. Prevention in the workplace focuses on minimizing the burden of workplace-related diseases and associated risk factors [21,22]. WHPP interventions range from behavioral measures with active participation (eg, courses or seminars) to organizational measures such as consultations, analyses, inspections, and establishing organizational structures such as a health committee [23,24].

### Prior Work

With the Luxembourg declaration, WHPP has evolved into an independent discipline that differentiates from return-to-work (RTW) and occupational safety and health (OSH) measures [20,25]. In OSH-related disciplines, previous reviews have focused on risk assessment or detection related to physical ergonomics [26], occupational physical fatigue [27], or core body temperature [28]. Other reviews explored the evidence of AI in F-related areas, such as vocational rehabilitation [29] and functional capacity evaluation [30]. In health promotion in general, 1 review evaluates the use of chatbots to increase health-related behavior but does not focus on the workplace setting [31]. To the authors’ knowledge, no review has evaluated the use of AI in WHPP.

Therefore, this systematic scoping review aims to comprehensively assess an overview of the current use of AI in WHPP. The results will then be used to point to future research directions. The following research questions (RQ) were derived from these aims:

- RQ1: What are the study characteristics of studies on AI algorithms and technologies in WHPP?
- RQ2: What specific WHPP fields (prevention, behavioral, and organizational approaches) are addressed by the AI algorithms and technologies?
- RQ3: What kind of interventions were conducted, and what outcomes were assessed?

## Methods

### Design

A systematic scoping review approach [32] was selected following the extended PRISMA-ScR (Preferred Reporting Items for Systematic Reviews and Meta-Analyses extension for Scoping Reviews; Multimedia Appendix 1) [33]. We applied the 5-step framework to identify current or emerging research directions and provide an overview of research activities [34]. Additionally, the patient or population, intervention, comparison, and outcomes (PICO) framework [35] was used to specify the study’s objective, from the search string and data charting to more systematic discussion [36]. The review was registered prospectively in the Open Science Framework (OSF) on July 5, 2023. All files (protocol, search string, and search results) have been uploaded to the OSF profile and are publicly accessible [37].

### Eligibility Criteria

Included studies needed to be (1) peer-reviewed, (2) written in English, and (3) focused on any AI-based algorithm or technology that (4) were conducted in the context of WHPP, or (5) an associated field (workplace prevention, occupational health, and workplace health) that applies to WHPP. The types of research considered were review types (systematic, scoping, or rapid), cross-sectional studies, and longitudinal studies.

Our conceptualization of AI included the concepts of “machine learning,” “deep learning,” and “natural language processing.” Our conceptualization of “workplace health promotion and prevention” followed a broader understanding comprising the setting (eg, “work,” “workplace,” or “in or at the workplace”), the target population (eg, “working adults” or “employees”) and the outcome dimension (eg, “health” or “health behavior”). The search period was limited to studies published since January 2000 and before July 31, 2023. During the review, the search was extended to December 20, 2023.

### Information Sources and Search

The systematic literature research was conducted in July 2023 in 3 databases: PubMed, IEEE Xplore, and Association for Computing Machinery. The search string included Boolean operators (“AND,” “OR,” and “NOT”) and search terms related to “artificial intelligence,” “workplace health promotion,” “health promotion,” and “workplace setting” (see supplementary files available at OSF profile [37]). Papers were managed with the software tool Rayyan, followed by a 2-stage screening process. First, 1 reviewer (ML) removed all duplicates. Second, 2 reviewers (ML and AL) screened all titles or abstracts and read full texts for eligibility criteria in a blinded procedure. Disagreement was resolved by either consensus of the 2 reviewers or by consultation of a third reviewer (IK).

### Data Charting and Synthesis of Results

In the first step, the study characteristics were extracted: first author (name and year), study design (eg, cross-sectional or randomized controlled trial), the primary type of AI algorithm and technology as referred to in the study (eg, AI, ML, DL, or NLP), and the frontend in which the AI-technology was implemented (eg, mobile app or web app). Second, the PICO framework [35] was applied to extract information about the target group (number of included participants/workplace context), the intervention approach, the comparison, and the reported outcomes of the study.

We used the extracted information from the study characteristics to answer RQ1 on current AI-based technologies applied in WHPP. For answering RQ2 and RQ3, we used the data extracted by the PICO framework. The information was then categorized within the results’ tables and summarized narratively.

## Results

### Included Studies

**Figure 1:**
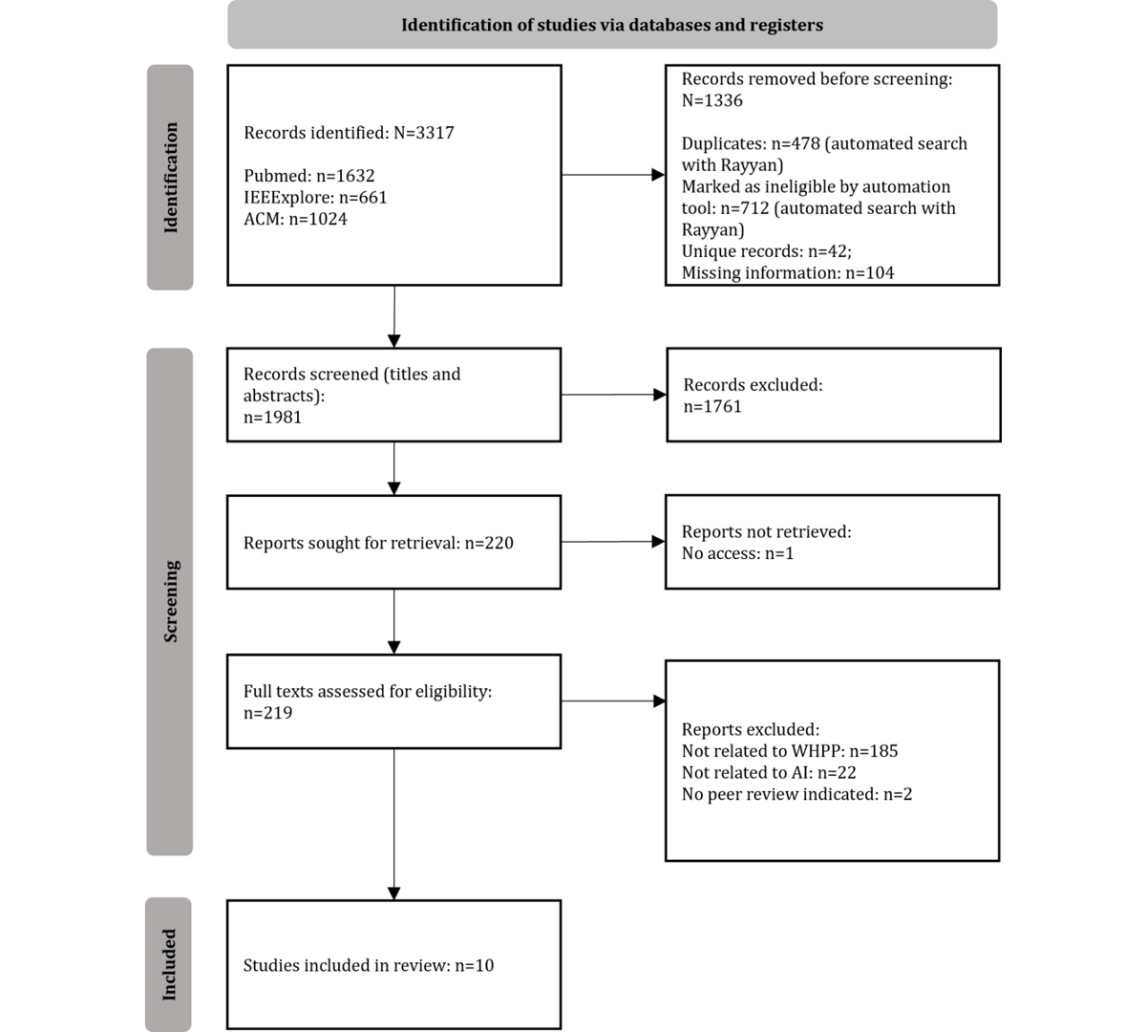
PRISMA flowchart of the literature search process. ACM: Association for Computing Machinery; AI: artificial intelligence; PRISMA: Preferred Reporting Items for Systematic Reviews and Meta-Analyses; WHPP: workplace health promotion and prevention.

The predefined search led to a total of 3317 results. The screening results revealed 478 duplicates, 712 records not meeting inclusion criteria (eg, publication type, language, or setting), 42 unique records, and 104 with missing information, leaving 1981 records for the title and abstract screening. The title and abstract screening excluded another 1761 records for not meeting inclusion criteria, leading to 220 records for full-text screening, of which one was inaccessible. After screening 219 full-text records, another 209 records were excluded. Finally, 10 studies remained in this systematic scoping review (the PRISMA-ScR flowchart is shown in Figure 1).

### Study Characteristics (RQ1)

The results of the study characteristics are presented in Table 1. Regarding the study designs, 6 studies were cross-sectional studies [38-43], 3 were randomized controlled trials [44-46], and 1 was a quasi-controlled trial [47]. None of the studies explained data protection standards (security protocols, storage location or duration, or access of third parties) within the AI algorithms and technologies used. In most studies, white-collar workers were the intended target group [38,41,42,46], whereas, in 3 studies, white-collar and physical labor workers participated [40,45,47]. Further, 1 study evaluated AI-based technologies with physical labor workers [39], and another did not disclose any information about the type of work setting [44]. Information on sample characteristics was missing in 3 studies [40,41,44], little information was provided in 2 studies [38,44], and 4 studies offered sufficient information [39,42].

A comparison was used in different ways by 6 studies [40,42,44-47]. Further, 4 studies recruited a classic control group [39,44,46,47], 2 of which exposed the control group after a waiting period [44,46]. Another study compared their assessed data to external data thresholds [40], and 1 study compared assessed objective data with subjective data [42]. Regarding the outcome, all studies stated sufficient and significant results. Further, 1 study reported no changes in 1 of the 3 assessed outcomes [47].

**Table 1:** Study characteristics, AI^a^ algorithms and technologies, and WHPP^b^ fields.

Author	Year	Included type of AI algorithm	Implemented frontend	WHPP field	Study design
Anan et al [45]	2021	Machine learning	Smartphone app with integrated chatbot	Prevention; behavioral health promotion	RCT^c^
Morshed et al [38]	2022	Machine learning	Software-based sensor technology	Prevention	CS^d^
Cui et al [39]	2020	Deep learning networks (recurrent neural network or long-short-term neural network)	N/A^e^	Prevention (risk assessment)	CS
Dijkhuis et al [44]	2018	Machine learning	Web app	Behavioral health promotion	RCT
Hungerbuehler et al [40]	2021	Machine learning	Viki chatbot within a web browser interface	Prevention (risk assessment)	CS
Kaiser et al [41]	2021	Fuzzy neural network-based fusion	Smartphone app with GPS^f^ and eHealth sensor	Organizational health promotion (risk assessment)	CS
Lopes et al [47]	2023	Neural language processing or machine learning	EMYS^g^ robot	Behavioral health promotion	qCT^h^
Maxhuni et al [42]	2021	Machine learning	Smartphone app	Prevention (risk assessment)	CS
Piao et al [46]	2020	Deep learning networks, machine learning, and natural language processing (large language model)	Watson conversation tool (IBM Corp) integrated into a smartphone app	Behavioral health promotion	RCT
Yan et al [43]	2020	Convolutional neural network	Web-based app	Prevention (risk assessment)	CS

^a^AI: artificial intelligence.

^b^WHPP: workplace health promotion and prevention.

^c^RCT: randomized controlled trial.

^d^CS: cross-sectional study design.

^e^N/A: not applicable.

^f^GPS: Global Positioning System.

^g^EMYS: emotive head system.

^h^qCT: quasi controlled trial.

### AI Applications and Technologies in Specific WHPP Fields (RQ2)

AI algorithms and technologies were mainly used for preventive purposes in risk assessment (Table 1). Furthermore, 2 studies evaluated prediction models [39,42]. Additionally, 3 studies [44,46,47] targeted health behavior change using 3 different approaches ranging from a web app [44] and smartphone app [46] to social robot agents [47]. Further, 1 study [41] was categorized as an organizational health promotion approach. A major target indication was mental health, which was addressed in 4 studies [38,40,42,43]. In contrast, 1 study dealt with musculoskeletal disorders [45] and 1 on overall physical health and work-related factors [39].

### Interventions and Outcomes (RQ3)

The PICO category “intervention” did not apply to studies focusing on prevention since they did not evaluate an intervention [38-43]. Interventions were evaluated by 4 studies [44-47] with a duration of 12 weeks [44-46] and 8 weeks [47]. Within these 4 studies, 2 used chatbots as a primary AI application [45,46], 1 used a web application [44], and 1 used a social robot agent [47]. These 4 studies recruited a control group, of which 2 studies exposed the control group after a waiting period [44,46]. Regarding the outcome, all studies stated sufficient and significant results. The study of Lopes et al [47] reported no changes in 1 of the 3 assessed outcomes (Table 2).

**Table 2:** Interventions and outcomes of studies included in the review.

	Population	Intervention	Comparison	Outcome
Anan et al [45]	IG^a^ 48 and CG^b^ 46 engineers and white-collar workers	AI^c^-assisted program for MSD^d^ that selects exercises depending on participants’ chat input; 12-week intervention with individualized exercises for stretching, maintaining good posture, and mindfulness.	CG: exercise routine of 3 minutes per day during break time; routine consists of standard exercises for stretching, maintaining good posture, and mindfulness.	Adherence rate: 92%; significant difference in the worst pain scores of neck or shoulder pain or stiffness and low back pain between baseline and 12 weeks (score: –1.12; 95% CI –1.53 to –0.70; *P*<.001); significant improvements of IG in the severity of the neck or shoulder pain or stiffness and low back pain compared to CG (OR^e^ 6.36, 95% CI 2.57-15.73; *P*<.001); subjective improvement in symptoms in IG at 12 weeks (score: 43; 95% CI 11.25-164.28; *P*<.001).
Morshed et al [38]	46 remote information workers	Development and implementation of a workplace stress sensing system for 4 weeks using passive sensors (email, calendar, app, mouse and keyboard use; facial positions and facial action units; or physiological sensors).	Comparison of passive sensor data with self-report (study intake, experience sampling, daily check-in, daily check-out, end of study expectations) data.	Passive sensors detect triggers and manifestations of workplace stress effectively (eg, keyboard activity and less facial movement were positively correlated with stress (*r*=0.05, *P*<.05^f^ and *r*=0.09, *P*<.05^f^, respectively); the quality of stress models depends on prior data of the worker and the amount of data (*F*_1_-score: after 10 days=58%; after 19 days=73%).
Cui et al [39]	4000 steel workers	Development and comparison of 2 AI-based risk prediction models (LSTM^g^ vs RNN^h^) that predict the influence of the work environment on employees’ health.	N/A^i^	Based on sociodemographic data (age, income, education, or marital status), health-related data (BMI, smoking, drinking, or blood lipids [cholesterol or triglyceride]), and work-related factors (length of service, high-temperature exposure, shift work, or noise exposure) the prediction effect of LSTM is significantly better than that of traditional RNN, with an accuracy of more than 95% (*F*_1_-score).
Dijkhuis et al [44]	IG 24 and CG 24 population/setting not disclosed	Development and implementation of a prediction model that personalizes physical activity recommendations. Within a 12-week workplace health promotion intervention. The goals of the intervention were to increase physical activity during workdays by improving physical and mental health and several work-related variables.	CG: no participation in the 12-week WHP^j^-program.	Input variables “hours of the day” and “step count” were used in the evaluated model and reached an accuracy of 90% (mean accuracy=0.93; range=0.88-0.99; mean *F*_1_-score=0.90; range=0.87-0.94). Tree algorithms and tree-based ensemble algorithms performed exceedingly well. The individualized algorithms allow for predicting physical activity during the day and provide the possibility to intervene with personalized feedback.
Hungerbuehler et al [40]	77 industrial, logistic, and office workers	Development of a chatbot system and its implementation in a workplace setting to assess employees’ mental health.	Participation rates were compared to face-to-face collection method rates.	The response rate was 64.2% (77/120). The majority scored in the mild range for anxiety (GAD-7^k^: mean 6.21, SD 4.56; 50%) and depression (PHQ-9^l^: mean 4.40, SD 5.21; 57%), the moderate range for stress (DASS-21^m^: mean 11.09, SD 7.13; 46%), subthreshold level for insomnia (ISI^n^: mean 9.26, SD 5.66; 70%), the low-risk burnout-category (OLBI^o^: mean 27.68, SD 8.38; 68%) and in the increased risk category for stress (JSS^p^: mean 32.38, SD 3.55; 69%). Chatbot-based workplace mental health assessment is highly engaging and effective among employees, with response rates comparable to face-to-face interviews.
Kaiser et al [41]	12 office workers	Evaluation of a portable health (pHealth) app to detect COVID-19 infection and trace movement to prevent further infections. Additionally, the pHealth app detects employees’ health conditions and recommends further health measures if indicated.	N/A	The app-integrated COVID-19 questionnaire was validated against real-time health conditions. Proximity detection, contact tracing, and health monitoring (external sensors) were confirmed by proximity testing (surf plot evaluation); it effectively estimates COVID-19 infection risk and personal health conditions.
Lopes et al [47]	IG 28 and CG 28 service and retail workers	IG interacted with a social robot agent that promotes health behavior change of participants’ choice (physical activity, nutrition, tobacco consumption, and stress and anxiety) in the workplace. After baseline assessment 8, social robots were used for 20-30 minutes weekly for 8 weeks. Based on the health action process approach model, the intervention focused on goal setting, monitoring behavior, elaborating action plans, and self-efficacy techniques through videos.	CG received the same intervention measures through human agents via Teams (Microsoft Corp).	IG improved significantly compared to CG in productivity (*F*_1,46_=9041, *P*<.005^f^; η2=0.26) and in well-being (*F*_1,53_=4517, *P*<.005^f^; η2=0.079), but not in work-engagement (*F*_1,49_=0.5176, *P*>.005^f^). Additionally, IG improved significantly in the postintervention scores compared to CG (*F*_1,43_=8997, *P*<.001^f^, Wilk Λ=0.597, partial η2=0.40) despite presenteeism and regard for their level of mental well-being.
Maxhuni et al [42]	30 office workers	Measurement of smartphone data to assess employees’ stress levels. Data were assessed for 8 weeks on physical activity (accelerometer), location (GPS^q^), social interaction (microphone, number of phone calls, or text messages), and social activity (app usage).	Objective data was compared to subjective data (OLBI, POMS^r^).	A high correlation between objective smartphone data and questionnaire scores was overall significant. The accuracy of the supervised decision tree was acceptable (*F*_1_-score=67.5%). The semisupervised learning approach was somewhat better, with an *F*_1_-score of 70%. Overall, the results confirm that the prediction model is feasible to detect perceived stress at work using smartphone-sensed data.
Piao et al [46]	IG 57 and CG 49 office and administrative workers	A healthy lifestyle coaching chatbot from the KakaoTalk App (Kakao Corp) was implemented into an office work setting to promote employees’ stair-climbing habits. During the intervention, the IG received cues, intrinsic, and extrinsic rewards for the entire 12 weeks.	CG did not receive intrinsic rewards for the first 4 weeks and only received all rewards, as in IG, from the fifth to the 12th week.	After 4 weeks, the change in SRHI^s^ scores was (mean IG 13.54, SD 14.99; mean CG 6.42, SD 9.42) significantly different between groups (*P*<.05^f^). Between the fifth and 12th week, the change in SRHI scores of the intervention and control groups was comparable (mean IG 12.08, SD 10.87; mean CG 15.88, SD 13.29; *P*=.21). Level of physical activity showed a significant difference between the groups after 12 weeks of intervention (*F*_1,11_=21.16; *P*=.045). Intrinsic reward was significantly influencing habit formation.
Yan et al [43]	352 respiratory therapists in medical centers and regional hospitals	Building a model to develop a web-based application for classifying mental illness at the workplace. Data on emotional labor and psychological health was assessed for 4 weeks with the ELMH^t^.	N/A	Model structure with 8 domains was confirmed with exploratory factor analysis, and 4 types of mental health were classified using the Rasch analysis with an accuracy rate of MNSQ^u^=0.92. An app predicting mental illness was successfully developed and demonstrated in this study.

^a^IG: intervention group.

^b^CG: control group.

^c^AI: artificial intelligence.

^d^MSD: musculoskeletal disorder.

^e^OR: odds ratio.

^f^Original *P* values were not reported in the original publications.

^g^LSTM: long short-term memory.

^h^RNN: recurrent neural network.

^i^N/A: not applicable.

^j^WHP: workplace health promotion.

^k^GAD-7: Generalized Anxiety Disorder Scale.

^l^PHQ-9: Physical Health Questionnaire.

^m^DASS-21: Depression, Anxiety, Stress Scale.

^n^ISI: Insomnia Severity Index.

^o^OLBI: Oldenburg Burnout Inventory.

^p^JSS: job strain survey.

^q^GPS: global positioning system.

^r^POMS: profile of mood states.

^s^SRHI: self-report habit index.

^t^ELMH: Emotional Labor and Mental Health questionnaire.

^u^MNSQ: mean square error.

## Discussion

### Principal Results

#### Overview

This study aimed to assess an overview of the current state of AI use in WHPP. Our results underline that despite the rapid increase in AI-related studies, only a small number of studies have addressed AI apps and technologies in WHPP up to now. Risk prediction and modeling were the most identified WHPP fields, followed by behavioral health promotion approaches. AI algorithms and technologies were primarily implemented in smartphone apps (eg, in the form of a chatbot) or used the smartphone as a data source (eg, GPS). Further, our results revealed that most studies validated AI algorithms and feasibility.

#### Potential Approaches

The results merely indicate the potential of AI in WHPP with individualized, real-time data analysis and health-related information as critical elements but do not fully reflect this at present. AI-assisted chatbot apps were a primary AI technology, reaching reasonable adherence rates and offering a potential access route through various frontend solutions such as smartphones or web-based apps. Chatbots can easily individualize health-related information and recommendations regarding the type of job, educational level, and specific language barriers. The integration of sensor technologies can increase the efficacy of individualized chatbot solutions. This could advance the access and dissemination of workplace health-related information significantly. Chronically ill employees or other target groups can profit from context-specific health information that helps maintain or improve workability [48]. The aspect of anonymity might increase the acceptance of prevention measures for smoking cessation, alcohol, or substance abuse [31,49]. Due to the diversity of job activities (eg, physical labor or white-collar jobs) and workplace characteristics (eg, office, hybrid, or remote work), individualized access to health interventions can improve resource allocation as well as the density and quality of preventive health care [50,51]. Personalizing health-related information or feedback potentially increases workplace health-related behaviors [52,53]. The genuine ability of AI to analyze large amounts of data in real-time can be applied to predict or detect individual or organizational health risks, for example, infections, stress symptoms, or body positions [54-59].

#### State of AI-Research in WHPP

The small number of studies on AI and WHPP compared to other sectors of work-related health (eg, OSH or RTW) or public health indicates a considerable research gap. At this point, research in other health care sectors offers much more reviews [7,60-62]. Reasons can be found in common challenges of WHPP as a young research field, a high sensitivity regarding data protection regulation in the context of work, and the nonexistent legal requirements for WHPP in many countries [23,63,64]. At the same time, WHPP is often entrenched within an OSH paradigm among employers that do not prioritize WHPP [65,66].

As stated, most research WHPP fields were prevention and risk prediction followed by behavioral approaches. Stress and mental health were the primary outcomes of 4 studies within these fields. Given the relevance of mental health, the research interest can be assessed as adequate. At the same time, musculoskeletal disorders are the leading cause of sick leave in most countries [67] and are therefore highly underrepresented in the included studies. In 2 studies, behavioral approaches focused on physical activity and general health behavior were investigated in 1 study. Other WHPP-related behaviors such as nutrition, sleep, substance abuse (eg, nicotine), or stress management are not targeted by current research [24]. The same accounts for organizational WHPP approaches centered in only 1 study [41]. Organizational approaches that aim to disseminate health-related information, increase work-related health literacy, or implement educational measures have not been included in current AI and WHPP research. Areas such as social inequality [68], specific target groups (eg, chronically ill employees or migrants), or health-oriented leadership were not addressed.

Most studies of our review were conducted in a cross-sectional study design to gain data for any AI learning process in a time- and resource-efficient way [69]. This has 2 implications regarding the current stage of research. First, AI model life cycles need to be completed to gain high-level semantics and create a comprehensive learning basis, from data preparation (eg, dealing with missing data) and data conditioning to data acquisition and model refinement [70]. For future AI models, longitudinal data are of utmost importance, as cross-sectional data can only reflect on a specific stage of that life cycle [70,71]. Second, longitudinal study designs are usually more cost- and resource-intensive and often less prioritized. This not only leads to an imbalance of evidence on behavioral WHPP interventions but also to a lack of causal relation between AI and WHPP outcomes.

Most studies reported using ML compared to more sophisticated DL or NLP algorithms. ML algorithms use extracted data to predict binary or multiple outcomes or classes without hidden layers. DL algorithms are characterized by hidden-layer neural networks. They can be employed for the analysis of more complex data sets, for example, for the detection of multidimensional objects in the realm of video and speech analysis [4,72]. The complexity of DL algorithms, in turn, ties in with the AI model life cycle, as DL algorithms require a broader database for learning. While ML approaches are found to be highly predictive and offer more individualized interventions in a specific context, they are also prone to errors. Escorpizio et al [29] point out that in 1 study, ML classification exceeded clinicians’ decision-making [73]. Still, the results were later reversed when the approach was implemented with a different cohort [74]. This is of particular interest, as studies within our results relied on either a small number of participants [41], few input variables [44], or a homogenous data input (eg, only self-report data) [40], causing potential ceiling effects within the AI learning progress [75,76]. Conversely, the benefit of longitudinal data in the context of AI reveals itself through the increase in precision. Further, 1 study pointed out the relevance of multiple measurements and longitudinal data by increasing the accuracy from 46% (time point 0) to 73% after 19 days of data [38]. Nevertheless, the included studies do not use the potential of AI in comparable health-related fields such as OSH or RTW [26-31]. Some areas of AI application are not addressed, such as big data analysis (eg, comparison with existing data of national cohort studies) or language translation models.

### Future Research

As pointed out, current research is on AI in WHPP regarding quantity, fields of WHPP and its subdomains, and AI algorithms. Future research should center around major causes of sick leave, such as musculoskeletal disorders, mental health, respiratory conditions, and influenza [67]. Behavioral WHPP interventions should extend to all areas of health-related behavior, including nutrition, sleep, substance abuse, and stress management [24]. Further, setting-specific aspects of WHPP, such as intervention content, implementation strategies, user experience, design, algorithms, and the company’s size, need to be considered more specifically. So far, the studies have provided only moderate information on the job activities or the target groups. At the same time, workplaces and workers are diverse. The health of employees is influenced by numerous organizational and individual factors that must be further considered in the learning cycle of AI [77-79]. Regarding potential errors, existing AI algorithms must be validated with different target groups [59,80], emphasizing the need for longitudinal data and its impact on learning algorithms [81,82]. Beyond this, the technological diversity of the presented studies opens new possibilities for target group-specific or individualized interventions. Providing health information to chronically ill employees, migrants with different language skills, or individualizing health topics of varying age groups can be provided more effectively through AI to move beyond a “one size fits” all paradigm [83,84].

Outside of the objective’s scope, we identified 2 aspects that can improve future research. First, the included studies reported overall positive results regarding feasibility, significance, or accuracy, underlining the vast potential that AI technology harbors. However, the results must be interpreted cautiously as certain information in the primary studies was not provided, assessed, or available at the stages of the investigated technology. For example, few studies mentioned a potential bias through the novelty [40,47] or the Hawthorne effect [45,47,85]. The novelty effect [86] applies to most of the included studies as they did not control for experience with new technologies or their affinity to them. Second, concerns about data access, storage or control, the ownership of AI-generated data, and its further use need to be clarified [87,88]. Standards should be derived and updated at appropriate intervals, especially new AI-generated knowledge based on employee’s personal information [89]. Transparency and high data protection regulation can increase adherence rates and reduce usage barriers [90]. In turn, we propose that future research should rely on reporting guidelines [76,91,92].

### Strength and Limitations

Of note, 1 strength of our review is the explanatory nature of the RQs and the systematic search strategy in this new field. Consequently, the heterogeneity of the identified studies might be considered a limitation. Different AI applications and technologies, the types of intervention, and the variety of workplace settings limit the conclusion significantly. Beyond this, the reporting of the types of AI-based algorithms and technologies used in the study are based on the authors’ self-reports. It is important to consider that the differentiation of the AI algorithm types cannot be made with a high degree of distinction.

### Conclusions

Overall, this review underlines that AI in WHPP bears considerable potential but is not used fully at present. The results of our review offer a promising perspective on the predictive and personalized health paradigm shift in WHPP. Nevertheless, we conclude that current AI-related research in WHPP is still at the beginning, as it does not cover the scope of WHPP. The most salient research gaps can be found in lacking fields of WHPP and its subdomains, the predominantly ML-based algorithms and cross-sectional data, and the weak consideration of the work context. We believe we have contributed to future WHPP research by identifying these gaps and recommending future approaches. As AI applications are gaining an increasingly important role, we are convinced that future research will profit from an extended range of research in all fields of WHPP, longitudinal data, and the use of reporting guidelines.

## Appendix

Multimedia Appendix 1 PRISMA-ScR (Preferred Reporting Items for Systematic Reviews and Meta-Analyses extension for Scoping Reviews) checklist.

## Abbreviations

AI: artificial intelligence
DL: deep learning
ML: machine learning
NLP: natural language processing
OSF: Open Science Framework
OSH: occupational safety and health
PICO: patient or population, intervention, comparison, and outcomes
PRISMA-ScR: Preferred Reporting Items for Systematic Reviews and Meta-Analyses extension for Scoping Reviews
RQ: research question
RTW: return-to-work
WHPP: workplace health promotion and prevention

## Data Availability

All data are publicly available in the OSF [37].
